# Mechanisms of Germline Stem Cell Competition across Species

**DOI:** 10.3390/life14101251

**Published:** 2024-10-01

**Authors:** Rachel A. Hodge, Erika A. Bach

**Affiliations:** Department of Biochemistry and Molecular Pharmacology, New York University Grossman School of Medicine, New York, NY 10016, USA; rachel.hodge@nyulangone.org

**Keywords:** cell competition, stem cell competition, germline stem cell, paternal age affect disorders, testis, ovary, mosaic analysis

## Abstract

In this review, we introduce the concept of cell competition, which occurs between heterogeneous neighboring cell populations. Cells with higher relative fitness become “winners” that outcompete cells of lower relative fitness (“losers”). We discuss the idea of super-competitors, mutant cells that expand at the expense of wild-type cells. Work on adult stem cells (ASCs) has revealed principles of neutral competition, wherein ASCs can be stochastically lost and replaced, and of biased competition, in which a winning ASC with a competitive advantage replaces its neighbors. Germline stem cells (GSCs) are ASCs that are uniquely endowed with the ability to produce gametes and, therefore, impact the next generation. Mechanisms of GSC competition have been elucidated by studies in *Drosophila* gonads, tunicates, and the mammalian testis. Competition between ASCs is thought to underlie various forms of cancer, including spermatocytic tumors in the human testis. Paternal age effect (PAE) disorders are caused by de novo mutations in human GSCs that increase their competitive ability and make them more likely to be inherited, leading to skeletal and craniofacial abnormalities in offspring. Given its widespread effects on human health, it is important to study GSC competition to elucidate how cells can become winners or losers.

## 1. Introduction

It is well known that individual organisms compete with one another, and organisms with traits best suited to their environment (higher fitness) will pass along their genetic material to the next generation, while less fit individuals will not. Mutations in the genome can augment or impair an individual’s fitness. Similarly, genetic heterogeneity between neighboring cells of the same type induces competitive interactions. Less fit cells (“losers”) are removed, and more robust cells (“winners”) are amplified. These loser cells are still viable when all cells in the tissue share their genotype [1,2]. Mutations that decrease translation, decrease proliferation, and/or alter proteostasis reduce fitness, creating loser cells that survive in a homotypic environment but are outcompeted by winner wild-type neighbors [3,4,5,6,7,8,9,10,11]. Winners have specific genetic advantages that allow them to eliminate losers. Mutations that enhance metabolism, increase proliferation, and/or kill neighboring loser cells increase cellular fitness, creating winner cells [12,13,14,15,16,17,18]. 

This phenomenon is referred to as cell competition, which was initially described in the *Drosophila melanogaster* imaginal wing disc, an immature larval tissue that differentiates into the adult wing [19]. A class of mutations in ribosomal proteins called *Minutes* [20,21] causes wing disc epithelial cells to be lost to apoptosis [3]. Gain-of-function mutation of the proto-oncogene *Myc* in the *Drosophila* wing disc can create “super-competitors” that expand at the expense of wild-type loser cells [22]. Myc-overexpressing winner cells have been found to kill wild-type loser cells up to eight cell diameters away [18]. When *Drosophila* wing disc cells autonomously overactivate the Wingless (Wg) pathway, a homolog of the mammalian Wnt pathway, these cells also kill wild-type neighbors [14]. Gain of function in other signaling pathways—such as JAK/STAT and Yorkie/YAP—also creates super-competitors [15,16,17]. Cell competition likewise occurs in developing mouse tissues. *Belly spot and tail (Bst)* was identified as a *Minute* gene in mice that similarly regulates competition during development [23]. Cells in the early mouse embryo were shown to be heterogeneous with respect to Myc levels, and cells with the highest relative levels of Myc (i.e., the winners) expanded at the expense of lower-Myc cells (i.e., the losers) without perturbing development [24]. 

Competition has been extensively documented between adult stem cells (ASCs), which exist throughout an individual’s life and continuously renew tissue. ASCs can undergo three types of cell division: asymmetric division, in which daughters with asymmetric fates are produced (one stem cell daughter and one differentiating daughter); symmetric differentiation, which produces two differentiating daughters; or symmetric renewal, which produces two stem cell daughters [25]. To renew tissue and simultaneously maintain their population, ASCs generally display population asymmetry, in which divisions are not restricted to the asymmetric outcome. ASCs reside in microenvironments called niches, which are necessary for ASCs to self-renew. Due to limited niche size and signal range, ASCs compete for niche access [26,27,28]. In homeostatic conditions, no individual ASC has an advantage over others; ASCs remain or are eliminated randomly, termed neutral competition [25]. Conversely, when one ASC becomes advantaged, it can remain in the niche at the expense of wild-type neighbors, referred to as biased competition [29,30,31,32]. 

Germline stem cells (GSCs) are ASCs that produce sperm and eggs. Unlike biased competition between somatic ASCs, which affects one tissue, biased competition between GSCs is more impactful, as it can alter the proportion of alleles transmitted to the next generation. In 1998, Otto and Hastings proposed the concept of “mitotic drive”, in which GSCs with a competitive advantage transmit their alleles above the expected 50% Mendelian rate [33]. This competitive advantage is provided by “selfish elements”, alleles that promote their own transmission at the expense of others. Mitotic drive is much less well characterized than its counterpart meiotic drive, wherein selfish elements are inherited at a super-Mendelian rate due to their influence on gametes [34,35,36,37,38,39,40].

GSC competition and, more broadly, ASC competition have critical effects on human diseases and disorders. While selfish elements could increase the likelihood that beneficial traits will be passed to the next generation, traits that are beneficial to germline cells are not necessarily beneficial to the resulting offspring. Selfish elements in human GSCs, called spermatogonial stem cells (SSCs), are thought to underlie paternal age effect (PAE) disorders. PAE disorders are a spectrum of spontaneous congenital disorders caused by de novo mutations (DNMs) in sperm. PAE-associated DNMs are correlated with increasing testis age and are thought to generate rare selfish SSCs that are positively selected and clonally expanded, possibly by outcompeting wild-type SSCs. All known PAE disorders are associated with dominant gain-of-function mutations in the receptor tyrosine kinase (RTK)-RAS-MAP kinase (MAPK) pathway, which is considered to be the most commonly mutated pathway in cancer [41]; for example, 95% of pancreatic cancers have activating mutations in *KRAS* [42]. PAE disorders include severe phenotypes such as congenital skeletal abnormalities, cardiac defects, and cancer predisposition [43]. Additionally, mutations in SSCs can generate spermatocytic tumors (SpTs), a rare form of cancer typically found in older men. Stem cell competition has also been broadly linked to tumorigenesis [44,45,46]. Therefore, uncovering mechanisms of stem cell competition is essential for improving a variety of health outcomes. 

Here, we discuss the history of GSC competition in *Drosophila*, tunicates, and mammals. We first describe the earliest studies of GSC competition. We then introduce the tunicate and *Drosophila* gonads as models to elucidate GSC competition mechanisms. Next, we summarize studies of competition between somatic ASCs, including their associated signaling pathways. We discuss the role of GSC competition in the formation of rare SpTs, as well as the role of somatic ASC competition in age-related disease. Finally, we describe PAE disorders and their hypothesized links to GSC competition. Genes involved in ASC competition and genes that link ASC competition with cancer are summarized in Table 1 and Table 2, respectively.

## 2. Early Studies of Germline Stem Cell Competition

Studies of germline selection after irradiation laid the foundation for uncovering the mechanisms of germline stem cell competition. A key study published in 1929 suggested that germline cell selection contributes to differing induced mutation frequencies across male germline stages [47]. Researchers in the field then debated in which germline stage the selection was occurring [48,49,50]. In 1966, Abrahamson and colleagues compared the frequency of X-chromosome lethal mutations to autosome lethal mutations in the male germline after irradiation. They reported the recovery of more autosomal lethal mutations, indicating that X-chromosome lethal mutations were selected against. Having only one X chromosome, X-linked recessive lethal mutations in males are not rescued by a second X chromosome as they are in females. They also found that selection was stronger in pre-meiotic cells (which includes GSCs) than in post-meiotic cells [51]. This study set the stage for clonal analysis studies to confirm the number and function of female GSCs [52]. 

**Figure 1:**
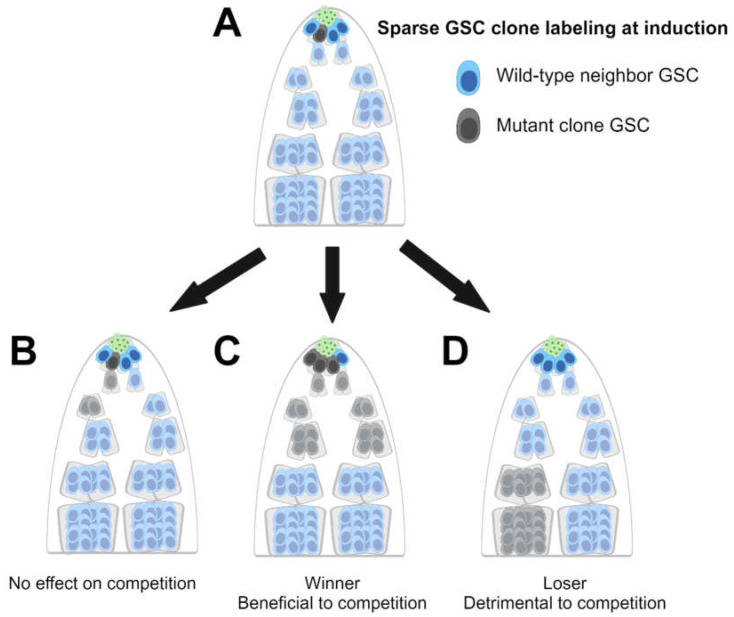
Clonal analysis for germline stem cell (GSC) competition-related genes in the *Drosophila* testis: (**A**) Marked GSC clones (dark grey) that are either wild-type or mutant for the gene of interest are sparsely induced in testes. Wild-type GSCs are dark blue, niche cells are green, and differentiating wild-type or mutant germ cells are light blue or light grey, respectively. In this example, 25% (1/4) of GSCs are induced to be marked as GSC clones. After time has passed, allowing the clones to proliferate, the testes are dissected and examined via microscopy. (**B**) If GSC mutant clones are present in the same proportion as when they were induced (25%), the gene is concluded to have no effect on competition. (**C**) If the number of GSC mutant clones increases relative to the number of wild-type unmarked GSCs (75% in this example), the mutants are winners, and the mutation is concluded to benefit the cell in competitive interactions. (**D**) If the number of GSC mutant clones decreases relative to the number of wild-type unmarked GSCs (0% in this example), the mutants are losers, and the mutation is concluded to be detrimental to the cell during competitive interactions. Created with BioRender.com.

In clonal analysis, individual clones are generated via mitotic recombination, demonstrated here in a model of the *Drosophila* testis (Figure 1) [53], but this technique has been used in other systems, like female GSCs in the ovary. Clones are either homozygous for a mutation in the gene of interest or wild-type for the gene of interest. All clones have a marker that is identifiable by microscopy. Labeling is induced sparsely so that the individual labeled cells and their progeny (“clones”) remain distinguishable and can be tracked over time. Clonal analyses reveal whether a gene plays a role in proliferation or survival. This is an invaluable tool for studying cell competition: if a mutant clone proliferates more or survives longer than its wild-type neighbors, this indicates that the mutation confers increased fitness and a competitive advantage. Conversely, elimination of a mutant clone over time suggests decreased fitness and a competitive disadvantage. Finally, if a mutant clone’s proliferation and survival are equal to those of its wild-type neighbors, this suggests that the mutated gene does not play a role in competition. Clonal analyses have demonstrated that germline selection can cause mutant female germ cell clones to be larger than wild-type clones [54] or can cause wild-type female germ cell clones to be larger than mutant clones that are heterozygous for loss-of-function mutations [55].

**Figure 2:**
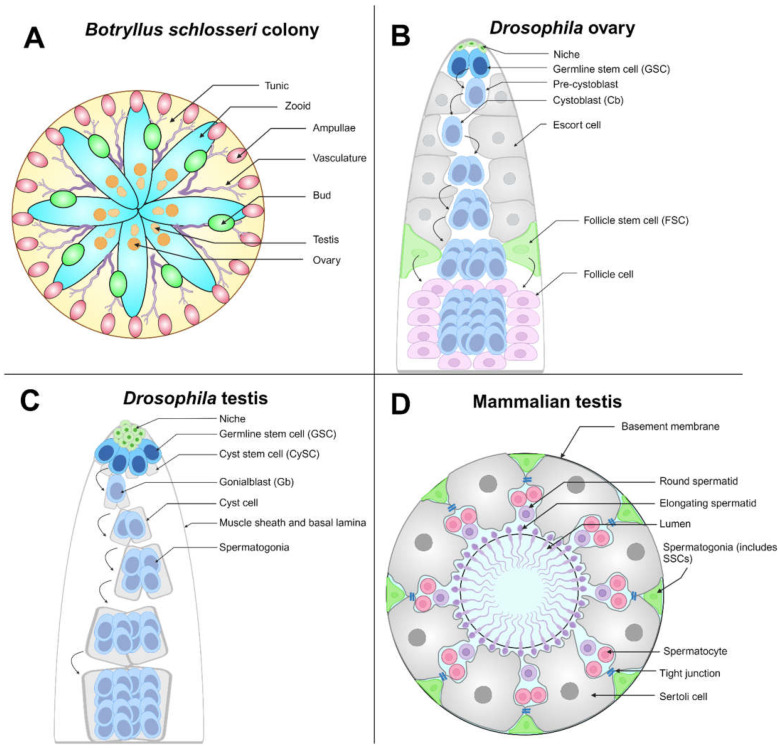
Germline stem cell (GSC) competition models: (**A**) *Botryllus schlosseri* are tunicates that can exist as a colony of zooids (as shown), with an outer covering called a tunic (yellow). Zooids (cyan) in the same colony are connected by their shared vasculature (purple). Colonies may reproduce asexually (forming buds, green) or sexually. The terminal ends of the vasculature, called ampullae (pink), may make physical contact with ampullae from other colonies, triggering a potential fusion of the two colonies. The depicted colony is hermaphroditic, having both ovaries and testes (orange). (**B**) The *Drosophila* ovary is linearly arranged, with niche cells (green) residing at the apical tip; 2–3 GSCs (blue) are in physical contact with the niche and undergo asymmetric division to generate a pre-cystoblast (light blue), which further matures into a cystoblast. The cystoblast differentiates, which requires the presence of escort cells (gray), and undergoes multiple incomplete cell divisions until a 16-cell germline unit called a cyst is generated. Follicle stem cells (FSCs, green) generate follicle cells (light purple), necessary support cells that surround the 16-cell germline cyst. (**C**) The *Drosophila* testis is a coiled tube wrapped in a muscle sheath. Niche cells (green) reside at the tip of the tube, and the niche maintains the GSC (blue) and somatic cyst stem cell (CySC, gray) populations. GSCs undergo oriented mitosis to produce a daughter gonialblast (Gb, light blue). Gbs (light blue) are encapsulated in two cyst cells (light gray), daughters of CySCs that are necessary support cells. The germline cells continue to divide and differentiate within the cyst, becoming spermatogonia, spermatids (not shown in diagram), and finally mature sperm (not shown in diagram). (**D**) Cross-section of the seminiferous tubule, the site of spermatogenesis in the mammalian testis. Spermatogonial stem cells (SSCs) are sparsely distributed, with no markers to distinguish them from other spermatogonia (green). SSCs are included in the spermatogonial population. Sertoli cells (gray), the equivalent of CySCs in mammals, are necessary support cells for developing spermatogonia. They are connected by tight junctions (blue), creating the blood–testis barrier. Spermatogonia further divide and differentiate into spermatocytes (including primary and secondary) (pink), which undergo meiosis to generate haploid round spermatids (purple) and then elongating spermatids (purple) that localize to the seminiferous tubule lumen. Spermatids will differentiate further to generate mature spermatozoids (sperm, not shown in diagram). Created with BioRender.com.

A variety of competition models are used across tissue types and species, and each offers its own distinct insights. One of the most unique models of cell competition is the tunicate *Botryllus schlosseri* [56,57], a colonial ascidian (sea squirt) (Figure 2A). *Botryllus* sexual reproduction produces embryos that develop into a motile chordate larval stage, which metamorphoses into the adult stage (oozooid). Oozooids are immobile invertebrates, and they must attach to a surface. After metamorphosis, adults develop testes, followed by ovaries, making them sequential hermaphrodites [58]. Adults can asexually reproduce by budding once a week, creating a colony of genetically identical clones (zooids) in a rosette shape embedded in a “tunic”. Zooids share an extracorporeal vasculature but can function independently. They can be surgically removed to create subclones that continue to grow on their own, allowing individual strains to be easily maintained in the lab. GSCs in *Botryllus* are self-renewing and lineage-restricted, and they retain pluripotency throughout an individual’s life [59]. Uniquely among cell competition models, *Botryllus* GSCs are mobile and can move through the vasculature. In each weekly reproductive cycle, GSCs will either settle and differentiate to produce gametes or self-renew and migrate to the niche in developing buds [60].

**Figure 3:**
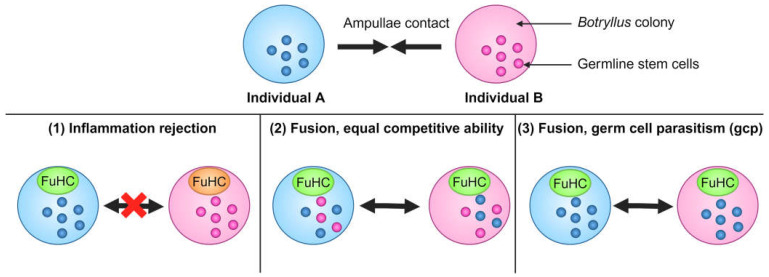
Germline stem cell (GSC) competition between *Botryllus schlosseri* colonies. Top: Two different *Botryllus* colonies can come into physical contact with one another via their ampullae. Bottom: There are three possible outcomes of this interaction: (1) The two colonies have incompatible *FuHc* alleles. An inflammation rejection response occurs, and there is no fusion. (2) The colonies have compatible *FuHc* alleles, and fusion occurs successfully. GSCs move between the colonies’ shared vasculature, and neither GSC lineage has a competitive advantage over the other, so both remain present in both colonies. (3) The colonies have compatible *FuHc* alleles, and fusion occurs successfully. GSCs move between the colonies’ shared vasculature, and Individual A’s GSC lineage (blue) has a competitive advantage over the other. Individual B’s lineage (pink) is outcompeted, and the GSC lineage in both colonies becomes monoclonal. This is termed germ cell parasitism (gcp). Created with BioRender.com.

When two individual colonies make physical contact via their ampullae, the terminal ends of their vasculature, it is possible for two ampullae to fuse, resulting in a parabiotic relationship (Figure 3) [61]. Alternatively, there may be an inflammatory rejection response, where no fusion occurs. Whether fusion can occur is dependent on the genetic locus *FuHc* (fusion-histocompatibility): individuals fuse if they express at least one of the same *FuHc* alleles [62]. Fusion results in GSCs migrating between individuals via the vasculature, resulting in potential GSC competition. If both individuals have GSCs of equal competitive ability, both lineages contribute to gamete production. If one individual has GSCs that can outcompete the other, the “winner” lineage will produce gametes in both individuals, and the “loser” lineage will be eliminated [56,57]. When one GSC lineage completely replaces another, this is referred to as germ cell parasitism (gcp), resulting in monoclonality of the winning GSC lineage in both colonies [56,63]. Clear hierarchies of colonies can be elucidated even when three colonies are fused to form a trichimera [64]. It has been hypothesized that the inflammatory reaction restricting parabiosis to kin, as well as the *FuHc* locus being highly polymorphic, helps prevent a single predatory GSC line from overtaking the entire species [63].

Studies of winner *Botryllus* GSCs have provided insights into possible mechanisms of competition. A recent study found that winner GSCs migrate through the vasculature faster, migrate in larger clusters, and have an advantage in niche occupancy compared with loser GSCs [65]. The advantage of larger cluster size is dependent on expression of the Notch ligand Jagged, and elevating Jagged expression in loser GSCs converts them into winners. Conversely, inhibition of the MAPK pathway converts winners into losers [65]. These findings indicate that the molecular mechanisms of GSC competition can be uncovered using *Botryllus* as a model system.

## 3. The *Drosophila* Germline Has Elucidated Cell Competition Mechanisms

*Drosophila* ovaries are composed of 12–16 ovarioles, and each ovariole contains a germarium (Figure 2B) and multiple egg chambers [66,67]. The ovary is linearly arranged: the germarium, which contains the resident stem cell populations, is at the proximal end, while the most mature egg chamber is at the distal end. Germaria contain 2–3 GSCs that are in physical contact with the niche at the germarium tip. GSCs divide asymmetrically to produce one GSC daughter cell and one pre-cystoblast daughter cell, the latter of which becomes a cystoblast (CB) cell upon induction of transcription of the differentiation-promoting gene *bag of marbles* (*bam*) [68]. CBs differentiate as they move away from the tip, dividing four times while encysted by somatic support cells called escort cells. Upon reaching the 16-cell cyst stage, germ cells are surrounded by another type of somatic support cell called follicle cells. These are generated by a resident population of follicle stem cells (FSCs). The germ cells ensheathed by follicle cells become egg chambers, and the produced oocytes differentiate into eggs [69]. Molecular and anatomical markers exist for all cell types in the fly ovary (and the fly testis; see below). Stem cell competition in the fly gonads can be robustly studied using clonal and quantitative assays at single-cell resolution. Powerful genetic tools are available in *Drosophila*, including mosaic clonal analyses and transgenic RNAi lines targeting nearly all of the 15,000 *Drosophila* genes [70,71,72,73,74,75,76,77].

GSCs in the *Drosophila* ovary compete with one another for physical access to the niche, and they will fail to self-renew if they lose contact [78]. Therefore, the *Drosophila* ovary has been well utilized as a model of GSC competition. Mutations in the differentiation-promoting genes *bam* or *bgcn* (*benign gonial cell neoplasm*) cause accumulation of undifferentiated GSC-like cells, which outcompete wild-type GSCs [68,79,80,81,82,83] (Table 1). 

**Figure 4:**
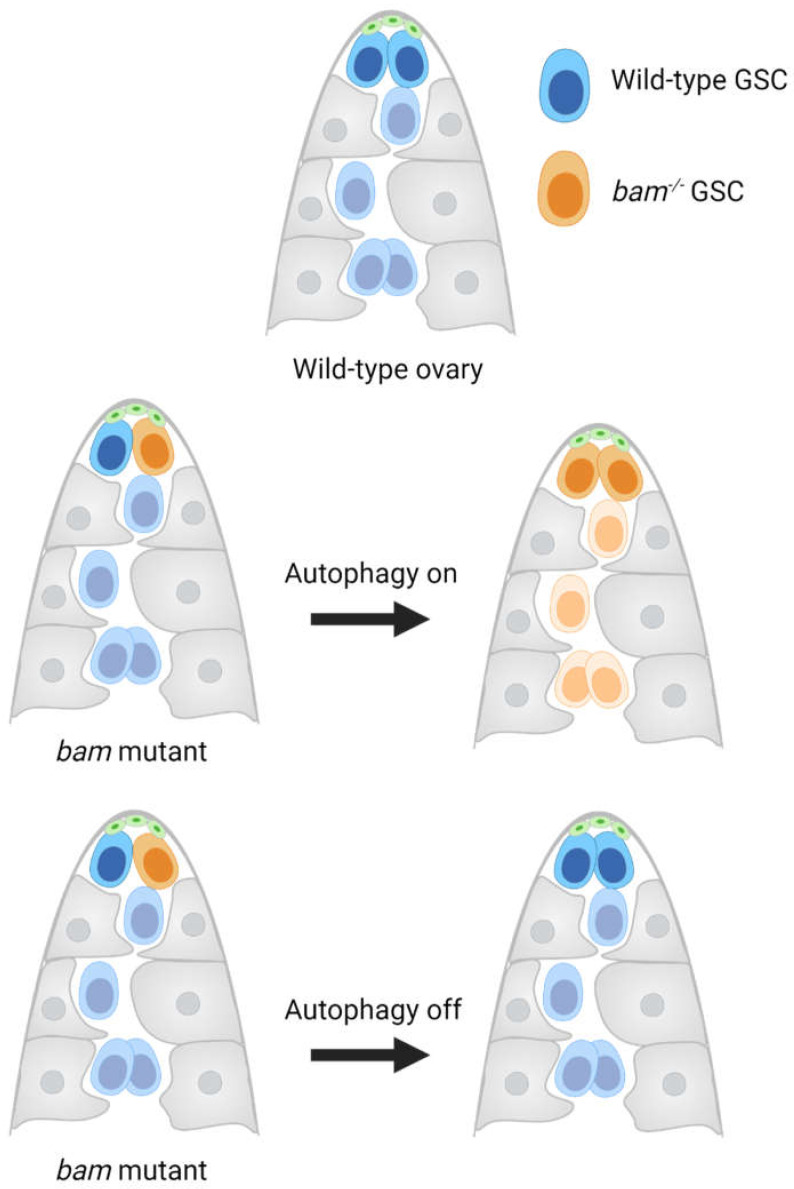
Germline stem cell (GSC) competition in the ovary. Mutation of *bag of marbles* (*bam*) in a female *Drosophila* GSC clone (orange) causes an accumulation of undifferentiated GSC-like cells (orange), which outcompete wild-type GSC neighbors (blue) for niche (green) access. Autophagy is required for *bam*-mutant germline cells’ competitive advantage; *bam*-mutant GSCs that have inhibited autophagy no longer outcompete wild-type GSCs. Escort cells are shown in gray. Created with BioRender.com.

The *bam*- or *bgcn*-mutant GSCs upregulate the adhesion protein E-cadherin to push wild-type neighbors out of the niche [81], though this upregulation may not be as crucial to their competitive advantage as initially thought [82,83]. Additional studies have found that *bam*-mutant GSCs upregulate autophagy [82] (Figure 4). Autophagy is required for their competitive advantage, and blocking autophagy in *bam*-mutant GSCs attenuates their cell cycle. Additionally, *bam*-mutant GSCs’ competitive advantage is further enhanced by starvation conditions, which are known to induce autophagy in the female germline [82,84]. Their cell cycle can also be attenuated by the loss of *insulin-like receptor* (*inr*), which encodes an upstream activator of the cell growth pathway mTOR, suggesting that *bam*-mutant GSCs rely on autophagy for cell proliferation. This contrasts with wild-type GSCs, which have low levels of autophagy [82]. Recent work has shown that *bam*-mutant GSCs have an accelerated cell cycle, which accounts in part for their ability to outcompete neighbors [83]. 

Increased expression of *Drosophila* Myc also causes female GSCs to outcompete their neighbors, which are expelled from the niche and differentiate [85] (Table 1). The authors of this study proposed that this competitive advantage is due to GSCs with higher Myc becoming more sensitive to Decapentaplegic (Dpp), a BMP pathway ligand that is secreted by the niche to promote stem cell renewal [85]. However, Jin et al. (2008) reported that *Myc*-null GSCs were not outcompeted [81]. Thus, the role of Myc in female GSC competition is still unresolved. 

The *Drosophila* testis is an ideal model to study stem cell competition (Figure 2C). The niche supports two stem cell populations: GSCs that produce sperm, and somatic cyst stem cells (CySCs) that are the functional equivalent of mammalian Sertoli cells [86]. Like their female counterparts, male GSCs adhere to niche cells [87,88,89]. GSCs continuously divide to produce a GSC daughter cell and a gonialblast (Gb) daughter cell, the latter of which undergoes transit-amplifying incomplete divisions to produce spermatogonia, which differentiate into spermatids and, finally, mature sperm. 

*Drosophila* male GSCs have been used to model adult stem cells’ self-renewal and differentiation dynamics. In this system, GSCs are lost with age and slow their mitotic rate, but they are replaced efficiently [90]. Live imaging of the testis has revealed that 80% of GSCs divide with an asymmetric outcome to produce one GSC and one Gb [91], consistent with analyses of fixed tissue [88]. It has also been shown that 7% of GSCs undergo symmetric renewal, resulting in two GSC daughters, while 13% of GSCs undergo symmetric differentiation, resulting in two differentiating daughters. This system has demonstrated plasticity, as spermatogonia can de-differentiate to become GSCs; both symmetric renewal and de-differentiation are upregulated following substantial GSC loss [91]. Live imaging has been successfully used to investigate GSC behavior during homeostasis and regeneration, but not yet to analyze GSC dynamics during competition.

**Figure 5:**
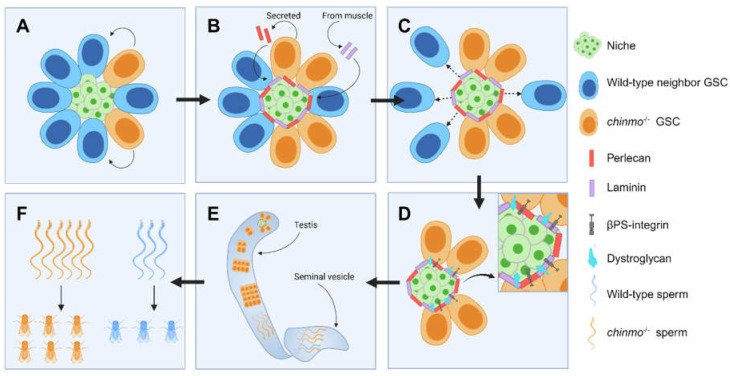
*chinmo*^−/−^ germline stem cell (GSC) clones (orange) outcompete wild-type GSC neighbors (blue) and take over the niche; arrows indicate time: (**A**) *chinmo*^−/−^ GSC clones are sparsely induced. (**B**) *chinmo*^−/−^ GSC clones form a moat around the testis niche (green) by secreting Perlecan (Pcan, red), resulting in the recruitment of Laminin (Lan, purple) from the nearby testis muscle sheath. (**C**) *chinmo*^−/−^ GSCs cause the expulsion of wild-type neighbors from the niche, (**D**) while remaining anchored to the niche via upregulation of Dystroglycan (Dg, cyan) and βPS-integrin (βPS, gray) (see inset). (**E**) Over time, the entire germline becomes monoclonal, (**F**) resulting in biased inheritance in offspring. Created with BioRender.com.

Most recently, we demonstrated that loss of the putative transcription factor *chinmo* from *Drosophila* male GSCs causes them to outcompete wild-type neighbors [92] (Figure 5 and Table 1). Surprisingly, this does not occur via mutant GSCs replacing their neighbors as do wild-type GSCs during adulthood [90] or wild-type intestinal stem cells (ISCs) [27] (see below). Additionally, competition by *chinmo*-mutant GSCs does not involve mechanisms of cell competition identified in the wing disc. Instead, *chinmo*-mutant GSCs secrete the heparin sulfate proteoglycan Perlecan (Pcan), which adheres to niche cells. This ectopic Pcan recruits another extracellular matrix (ECM) protein, Laminin (Lan), from the nearby basal lamina of the muscle sheath that surrounds the testis. These ectopic ECM proteins accumulate around the niche, forming a “moat”. While *chinmo*-mutant GSCs upregulate ECM-binding proteins (Dystroglycan (Dg) and βPS-integrin (βPS)) to remain in the niche, their wild-type neighbors do not and instead differentiate. This is notable because tumor-initiating cells have been shown to orchestrate ECM remodeling to promote tumor growth [93,94,95]. Over time, the germline becomes monoclonal, composed of only *chinmo*-mutant cells. We developed an assay to measure allele transmission in the F1 progeny and found that the *chinmo*-mutant allele was inherited at 65% (a super-Mendelian rate), compared to 50% for the *chinmo* wild-type allele. Thus, GSC competition can lead to biased inheritance, and these results were the first reported mechanistic evidence in support of the mitotic drive hypothesis [92]. Additionally, these results indicate that the *chinmo*-mutant allele acts as a selfish element. This framework predicts that any gene whose mutation in GSCs causes niche remodeling and selective retention of the mutant GSCs is acting as a selfish element. 

## 4. Somatic Adult Stem Cells Compete for Niche Access in Gonads

Numerous mechanisms of stem cell competition have been elucidated in several adult tissues, including the *Drosophila* testis [28,30,92,96,97,98,99]. Wild-type somatic stem cells (i.e., the CySCs) in the testis have been shown to conform to neutral drift dynamics, where a CySC can be lost and replaced stochastically by its neighbors [30]. Studies of CySC–CySC competition have demonstrated cases of mutant clones becoming either losers or winners. In the former case, any mutation that decreases fitness (e.g., reduces self-renewal or niche adhesion) will create a losing CySC. Indeed, CySC clones lacking the vesicle trafficking genes *Sec16^A^* or *shibere* become losers and are lost from the niche [100]. In the latter case, loss of the tumor suppressors *patched* (*ptc*) or *hippo* (*hpo*), which activate the Hedgehog (Hh) and Yorkie (Yki) pathways, respectively, makes CySCs into winners [30] (Table 1). Additionally, loss of the suppressor of cytokine signaling at 36E (Socs36E*)*, which represses epidermal growth factor receptor (Egfr)/Ras/MAPK signaling [28,30,96,101], or loss of Abelson (Abl) kinase [99] causes CySCs to become winners, indicating that several signaling pathways can control competition for niche access (Table 1). In these cases, winning CySCs exhibit biased competition, skewing normal behavioral dynamics in favor of the mutant cell [30]. Mechanistically, winning CySCs are advantaged via accelerated proliferation. Once the winning CySC and its descendants have taken over the somatic lineage, the CySCs begin to outcompete GSCs in a process termed CySC–GSC competition [28,30,96]. CySCs with loss of *ptc* or *hpo*, or with gain of Ras activity, cause a significant loss of GSCs through as-yet uncharacterized mechanisms [28,30,96]. 

The female counterpart of CySCs—the ovarian FSCs—also compete with each other for space in the germarium [102,103]. Clonal analyses and mathematical modeling have shown that FSCs conform to neutral drift dynamics, and that some mutations can bias competition in favor of the mutant FSC [104]. Additionally, gain of function in Hh, JAK/STAT, and Yki signaling generates FSC winners that outcompete wild-type FSCs [105,106] (Table 1). Like CySCs, some mutations that give rise to winning FSCs promote proliferation [106,107,108], suggesting that accelerated proliferation is a common mechanism for winning among somatic gonadal stem cells. 

## 5. Somatic Adult Stem Cells Compete for Niche Access in the Mammalian Intestine

Shifts in clonal dynamics have also been documented in other types of ASCs over time, with consequences for the genetic makeup of all cell types derived from them. For example, in the mammalian small intestine, ISCs reside at the base of intestinal crypts [109]. Daughter cells further divide and differentiate as they move out of the crypt and toward the villus tip [110]. Snippert et al. generated a multicolor Cre reporter dubbed the “confetti mouse”, which labels individual ISC clones. As the individual ages, each crypt drifts toward monoclonality, in which all cell types in the crypt are derived from a single ISC clone, as a result of neutral competition [27,111]. Another group contemporaneously published similar lineage tracing of ISCs in the mouse intestine [112]. Furthermore, ASCs from other mammalian tissues, including the human gut and human airways, also exhibit neutral competition [29,31,113,114]. 

Biased competition has been documented between ISCs. In vivo imaging of “confetti mice” revealed that ISCs positioned further from the center (base) of the crypt, and therefore further from the center of the niche, proliferated less than those closer to the center. As a result, “central” ISCs were more likely to survive (become winners), and “border” ISCs were more likely to be lost and replaced (become losers) [115]. This indicates that the proliferation rate can be a key factor in determining which ASCs become winners. 

## 6. Signaling Pathways in Somatic ASC Competition

Mouse models have established that stem cell competition can underlie tumorigenesis [44,45,46]. Vogelstein and colleagues identified 140 cancer-driving mutations, including *APC*, *EGFR*, *FGFR*, *HRAS*, *JAK1*, *JAK2*, *JAK3*, *MAPK*, *NOTCH*, *NRAS*, *PTCH1* (*Ptc* homolog), and *SOCS* (*Socs36E* homolog), that when mutated confer “super-competitor” status to the clone [116,117,118,119] (Table 2).

Tumor-initiating cells act as super-competitors, exemplified by *APC*-mutant ISCs causing colorectal cancers in mouse models [29,32,44,45,120]. Loss of *APC* upregulates the expression of *Notum*, a WNT target gene and negative regulator of WNT. Secretion of NOTUM by *APC*-mutant ISCs inhibits the proliferation of wild-type neighbors and causes them to differentiate (become losers) [44,45]. ISCs expressing a gain-of-function *KRAS* allele or mutant for *APC* proliferate faster and create monoclonal crypts faster than wild-type ISCs [29]. Similarly, human esophageal epithelial cells with the oncogenic *Pik3CA^H1047R/+^* mutation outcompete wild-type neighbors through cell fate biased toward proliferation [121], and human bone marrow cells with a gain-of-function mutation in *Jak2* outcompete neighbors via increased cell cycling [122] (Table 2).

It is important to note that while cell competition may be typically thought of as a driver of tumorigenesis, it also functions as a vital tumor suppressor. For example, epithelial defense against cancer (EDAC) refers to wild-type epithelial cells outcompeting neighbor cells expressing oncogenic *RasV12* [123], constitutively active YAP [124], or dominant-negative p53 [125] (Table 2). In the latter case, mutant p53 cells in the absence of wild-type cells will not be lost [125]. The role of YAP, part of the Hippo signaling pathway that can be upregulated in tumors, is particularly nuanced. While YAP expression in liver tumor cells drives their growth, having high YAP activity in these cells does not guarantee tumor progression. YAP is also upregulated in surrounding healthy hepatocytes, and when the YAP activity in healthy hepatocytes exceeds the YAP activity in tumors, the tumor cells are outcompeted. Thus, the relative level of YAP activity in neighboring populations determines whether the tumor grows or dies [126]. Similarly, in the thymus, T-cell progenitors are regularly turned over by competition between young and old T-cell progenitors, which have different gene expression profiles. In mice, loss of this competition causes T-cell acute lymphoblastic leukemia, which is derived from transformed T-cell progenitors [127]. Thus, it is of critical importance to identify and characterize genes that impart a competitive advantage or disadvantage to mutant cells that initiate or prevent tumorigenesis. 

## 7. Germline Stem Cell Competition Is Linked to Cancer

Spermatocytic tumors (SpT), previously referred to as spermatocytic seminomas, are a rare subset of testicular germ cell tumors (TGCTs) [128,129,130,131,132]. In the human testis, the site of human spermatogenesis is the seminiferous tubule (Figure 2D). SSCs are rare (in mice, 0.01–0.02% of cells in the seminiferous epithelium) [133,134]. There are no known markers to distinguish SSCs from spermatogonia. As spermatogonia divide and differentiate, they move away from the seminiferous tubule basement membrane and toward the lumen, and they are provided necessary support by Sertoli cells [135]. Within this system, the majority of TGCTs are derived from gonocytes, immature germline cells, and are mostly found in younger men. SpTs are slow-growing tumors that comprise about 1% of testicular tumors [128]. In contrast to other TGCTs, SpTs are derived from adult germ cells, likely spermatogonia, and are typically found in older men (median age: 54 years) [128,131,136,137]. Given these traits, it has been speculated that SpTs originate from selfish selection of spermatogonia with a competitive advantage in proliferation [138]. While SpTs are usually benign, 5–6% of cases have sarcomatous differentiation, which is associated with metastasis, resistance to treatment, and poor prognosis [139]. Despite the rarity of these cases, better understanding of the origins of SpTs will be important in future studies to improve the treatment of malignant SpTs.

## 8. Adult Stem Cell Competition Causes Age-Related Disease

In the human bone marrow, aged hematopoietic stem cells (HSCs) can experience clonal hematopoiesis, a condition where a mutant HSC clone represents a disproportionately high fraction of the total HSC population. It is estimated that 10–20% of people over the age of 70 have clonal hematopoiesis, which is a precondition for blood cancers and inflammatory diseases [140,141,142]. Surveys of mutations associated with hematological cancers revealed that nearly two-thirds came from mutations in *DNMT3A* (encoding a DNA methylase) and *TET2* (encoding a DNA methylcytosine dioxygenase). Mutations in *ASXL1* (encoding a chromatin regulator), *SF3B1, SRSF2, PRPF8,* and *U2AF1* (encoding splicing factors), as well as mutations in oncogenes and tumor suppressors, were also observed [143] (Table 1). 

When a cancer-associated mutation occurs in at least 4% of an individual’s nucleated blood cells without any clear disease, it is referred to as clonal hematopoiesis of indeterminate potential (CHIP). Studies of CHIP have elucidated multiple competition mechanisms. Mouse HSCs with mutations in *DNMT3A* or *TET2* are winners, as they outcompete wild-type HSCs in transplantation assays [144,145]. Human HSCs with *DNMT3A* or *TET2* mutations are more resistant to apoptosis induced by age-associated inflammation [146,147,148,149]. *DNMT3A*-mutant HSCs’ competitiveness is further enhanced by increased chromatin accessibility, leading to upregulation of growth-promoting transcription factors such as MYC [150]. Other genes have been shown to regulate HSC competition, but whose mutations are not correlated with CHIP. For example, mouse HSCs with mutations in the tumor suppressor Tp53 pathway outcompete wild-type HSCs [151] (Table 1). Separate studies have reported that *Tp53*-mutant HSCs will enter the cell cycle despite DNA damage, which may cause them to outcompete neighbors when cytotoxic drugs are used to treat cancer [152,153]. Additionally, activating mutations in the kinase JAK2*,* a JAK-STAT pathway component, causes increased HSC proliferation and, thus, clonal expansion [154] (Table 2). The HSC competition paradigm is utilized for HSC transplants for leukemia patients: healthy donor HSCs must outcompete diseased host stem cells to access the niche and become established [155].

## 9. Paternal Age Effect (PAE) Disorders Are a Negative Outcome of Germline Competition

Increased parental age is well known for its association with reduced fertility and increased risk to progeny’s health. Previous work has primarily focused on the role of maternal age in offspring health outcomes. For example, it has been well documented that increased maternal age is associated with decreased fertility, increased risk of chromosomal aneuploidies such as Down’s syndrome, increased risk of pregnancy complications, and increased risk of a range of disorders [156,157,158]. The role of paternal age is less well known, but increased paternal age has been linked to disorders like schizophrenia and autism, as well as poor outcomes for newborns such as low birth weight, low Apgar scores, and increased mortality [159,160,161,162,163]. Since the average age of fatherhood is increasing in the United States and other countries, it is important to characterize the health risks involved with advanced paternal age [41,164,165].

Mutations arise randomly and increase with age across all non-senescent cells due to errors during DNA replication. While this buildup of mutations over time in somatic cells can be consequential for the individual, including by contributing to tumorigenesis [166], mutations in somatic cells cannot be inherited by progeny. However, DNMs in SSCs are consequential for the next generation: sperm derived from a mutant germline cell will generate progeny with the same mutation in their somatic cells. Increased paternal age is associated with increased DNMs in the offspring’s genome [167,168,169,170]. This phenomenon has been historically considered unique to the male germline in mammals, since females produce all of their lifetime’s oocytes during fetal development. Mammalian oocytes arrest at the prophase I stage of meiosis until ovulation [171,172]; therefore, DNMs cannot accumulate in the female germline via pre-meiotic divisions over the lifetime. Conversely, SSCs divide continuously over the reproductive lifetime, providing more opportunities for mutations to arise [173]. However, it should be noted that a recent study indicated that short tandem repeat mutation rates in offspring increase with both higher maternal and paternal age, suggesting that DNA damage to quiescent oocytes can contribute to DNMs in offspring [174]. While DNMs can be derived from other sources, such as environment-derived DNA damage, SSC divisions are the primary source. As a result of this paradigm, approximately 80% of DNMs in offspring are paternally derived [173].

**Figure 6:**
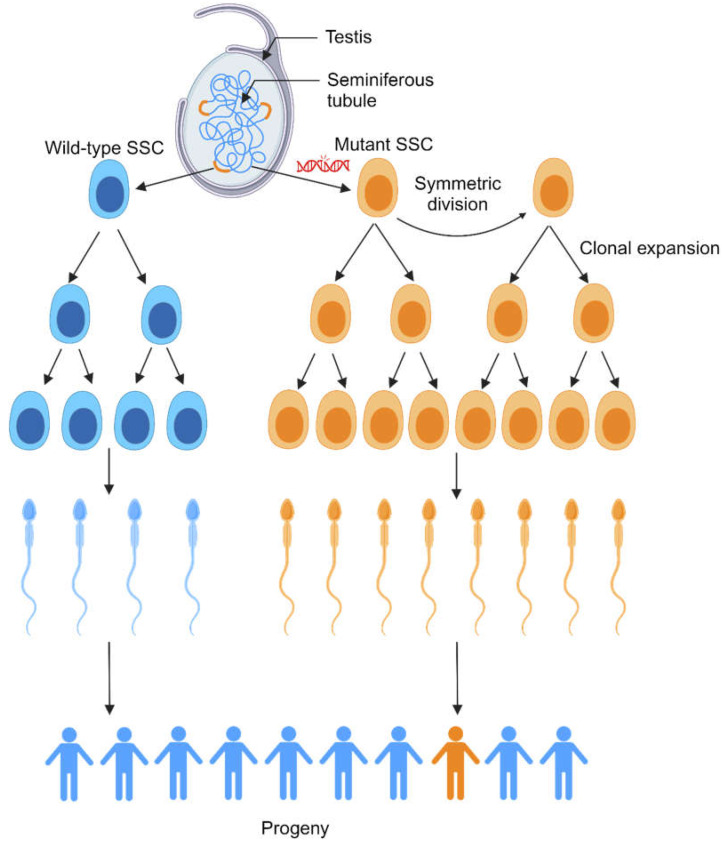
Paternal age effect (PAE) mutations result in clonal expansion of mutant spermatogonial stem cells (SSCs) (orange). Wild-type SSCs (blue) are sparsely distributed throughout the seminiferous tubules of the testis, and they divide to produce daughter cells that further differentiate to become mature sperm. SSCs mutant for PAE-associated genes undergo clonal expansion, leading to a much higher proportion of sperm produced per SSC than wild-type SSCs, possibly as a result of increased symmetric divisions. Therefore, a single SSC with a PAE-associated mutation is more likely to produce the sperm that ultimately fertilizes an egg than a single wild-type SSC. However, because PAE mutations are rare, the associated PAE disorders remain rare in progeny. Adapted from [41]. Created with BioRender.com.

Approximately 30–90 DNMs are passed to offspring [41,175], with DNMs from older fathers being at the higher end of this range. These DNMs may have a positive, negative, or neutral effect on the offspring’s fitness. Notably, a subset of DNMs are almost always derived from unaffected fathers, are associated with spontaneous single-gene disorders, and offspring are increasingly likely to have these disorders as paternal age increases [43,138]. These disorders typically are associated with craniofacial and skeletal abnormalities, and a subset are RASopathies [43], which are derived from mild gain-of-function mutations in RAS and can cause intellectual disabilities, congenital heart disease, increased cancer risk, and skin abnormalities [176]. All disorders derived from this DNM group have been dubbed paternal age effect (PAE) disorders [43] (Figure 6). All known PAE disorders arise from single gain-of-function mutations in the RTK-RAS-MAPK pathway [138,177]. Many are caused by mutations in several *FGFRs*, including *FGFR3* (achondroplasia) [178] and *FGFR2* (Apert [179], Crouzon, and Pfeiffer syndromes [180]). Others are caused by point mutations in *PTPN11* (Noonan syndrome) [181] and *HRAS* (Costello syndrome) [182] (Table 1). A sampling of human SpTs revealed a subset with mutations in *FGFR3* and *HRAS,* two PAE-associated mutations [129]. PAE-associated genes linked with SSCs’ self-renewal and differentiation have also been identified [138]. The SSC self-renewal gene *Glial cell line-derived neurotrophic factor* (*GDNF*) is associated with PAE disorders, and misexpression of *GDNF* produces malignant tumors in the testes that express germline markers [183] (Table 2). Additionally, deletion of the gene whose gain of function is associated with the PAE disorder Noonan syndrome, *PTPN11,* blocks differentiation of early germ cells [184]. While extensive aneuploidy appears to be the initiating event for SpTs [132], these studies together suggest that PAE-associated mutations may contribute to the progression of SpTs.

Strikingly, the likelihood of PAE disorders rises exponentially, not linearly, with paternal age [43,185]. This indicates that the linear increase in DNMs arising from SSCs with increased paternal age is not the sole contributor to PAE disorders. Further study showed that this exponential rise in likelihood with age is also due to selfish selection of germline cells with PAE-associated mutations. PAE-associated mutations, including those linked to RASopathies, appear to have gain-of-function properties that confer an advantage to SSCs, resulting in their clonal expansion [43,186]. A mathematical model of this clonal expansion suggests that SSCs mutant for PAE-associated genes occasionally undergo symmetric divisions to produce two SSCs, whereas wild-type SSCs exclusively divide asymmetrically to produce one SSC and one differentiating daughter cell [138]. As a result of presumably more SSCs mutant for PAE-associated genes, sperm carrying PAE-associated mutations are disproportionately represented in the sperm pool (up to 1000-fold higher than the baseline mutation rate) and are therefore individually more likely to be inherited by offspring than wild-type sperm or mutant sperm that do not have PAE mutations. Given that these gain-of-function mutations are in the RTK-RAS-MAPK pathway, the most frequently mutated pathway in cancer [41,187], the resulting clonal expansion has been compared to oncogenesis [43]. 

There are inherent challenges to studying PAE disorders and SSC competition in humans. PAE disorders are rare overall, as are DNMs in the germline, suggesting that there are extra protective mechanisms in place to maintain germline cells’ genomes [41]. Additionally, SSCs are distributed sparsely throughout the testes, and there are no definitive human SSC (hSSC)-specific markers. As a result, human studies to find the basis of PAE-associated mutant SSCs’ competitive advantage are particularly lacking. There are a small number of genes enriched in mouse SSCs (mSSCs), which have facilitated studies of SSC competition in mouse models [188]. Transplantation of fluorescence-activated cell sorting (FACS)-purified mSSCs into recipient mouse testes leads to competition between the transplanted and endogenous populations, and transplanted mSSCs are able to successfully colonize the testes in the long term, which could be utilized to study competition between mSSC populations [189]. Additionally, mSSCs appear to compete for fibroblast growth factors (FGFs) secreted by nearby lymphatic endothelial cells, which promote self-renewal. The mSSCs that consume lower amounts of FGF are outcompeted and differentiate. This is referred to as the “mitogen competition model” [190]. 

In vitro models of SSCs with PAE-associated mutations are also a possibility. Neonatal mouse, adult mouse, and adult human SSCs have been successfully cultured in the long term, although hSSCs cannot be efficiently expanded in culture, and their genetic stability in culture is unknown [188]. Such limitations need to be resolved before cultured hSSCs can be utilized for PAE disorder models, fertility treatments, or to select against sperm with deleterious mutations. Additionally, while mSSCs can be cultured more reliably, a possibly significant caveat to the mouse model of SSC competition was uncovered by Ryu et al. (2006); mSSCs from aged mice transplanted into young mice maintained their capacity for self-renewal and spermatogenesis for more than 3 years, well beyond the aged mouse’s natural lifespan [191]. This suggests that mSSCs do not acquire DNMs over time like hSSCs [188]. Nevertheless, mSSCs can be generated that express the Apert syndrome *FGFR2* mutation, and these mSSCs have increased competitiveness in in vitro models, as well as after transplantation [192]. 

## 10. Discussion

Cell competition occurs in a heterogeneous cell population and causes one subpopulation to become “losers”, which are eliminated, while “winners” remain. While cell competition is always beneficial to the winner cells, it can be beneficial or destructive to the tissue as a whole. Cell competition was initially characterized in the *Drosophila* wing disc [19], but in the intervening time it has been found across a variety of tissues and model systems [28,56,57,81,82,83,92,105,115,154]. In particular, the discovery of mechanisms that make cells into winners and losers is a broad and actively evolving field. For example, it was recently found that glutamate signaling, which is associated with cancer [193], regulates competition in the *Drosophila* wing disc, including *Myc*-related competition [194]. ASC competition is particularly consequential to human health because all cells in adult tissues that are regularly turned over are derived from ASCs. Within the ASC competition paradigm, cells with equal competitive ability are eliminated randomly, termed neutral competition; when cells have unequal competitive abilities and one group is eliminated, it is biased competition [27,29,30,112].

Models of somatic ASC competition have provided valuable insights into mechanisms of competition, and *Drosophila* has been an especially useful model to elucidate them. Studies of competition between *Drosophila* testis CySCs have revealed contexts where mutants may become winners or losers [28,30]. Notably, our understanding of the factors governing CySC–GSC competition and CySC–CySC competition is limited. Investigating CySC–GSC competition may provide a unique opportunity to improve our understanding of cell competition between different ASC types for access to the same niche. Similarly, *Drosophila* testis CySCs and ovary FSCs have been utilized in mathematical models to demonstrate neutral and biased competition dynamics [30,104]. In both systems, in vivo imaging could capture dynamics such as the way that loser stem cells are displaced from the niche. A robust in vivo imaging system has already been developed to monitor GSC behavior over extended time periods in the *Drosophila* testis [195]. The mammalian model of ISC competition has also been useful in demonstrating neutral drift (i.e., a shift in the genetic profile of the cell population in the absence of biased competition) toward monoclonality over time [27,111], as well as biased competition [115]. However, future studies could identify genes that confer winner status and identify the mechanisms that winners use.

ASC competition has been demonstrated to be involved in aging and cancer risk. HSCs in human bone marrow are increasingly likely with age to have mutant clones that are overrepresented in the population, known as clonal hematopoiesis. While this condition can exist in the absence of any known disease, it is associated with increased risk of blood cancers [140,141,142]. HSCs with cancer-associated mutations are able to outcompete their wild-type neighbors [152,153,154]. Given that global life expectancy is increasing [196], it will be important in the future to identify the mechanisms that can make HSCs into winners and find treatments to disrupt them. More broadly, cell competition has been demonstrated to play a role across many types of cancer. Many well-characterized cancer-driving mutations convert cells into super-competitors [116,117,118,119]. In some tissues, stem cells can acquire mutations that convert them to super-competitors, but once a tumor is formed, the tumor becomes heterogeneous due to genomic instability. This results in the gradual accumulation of mutations, followed by the emergence of subclones. Competition between tumor subclones can potentially select for winner clones that promote aggressive growth and metastasis [119]. Additionally, tumor subclones can cooperate to remodel the local environment [197,198,199]. One rare subtype of testicular tumor, SpT, may be uniquely demonstrative of germline cell competition. Given that SpTs are derived from early germ cells and are typically found in older men [128,131,136,137], it has been hypothesized that they are the product of SSCs with a competitive advantage [138]. Elucidating mechanisms of competition between tumor cells and healthy cells, as well as between heterogeneous tumor cells, will benefit our understanding of tumorigenesis and how cancers can become more aggressive over time.

While there is no animal model of SpT to characterize GSC competition, models in *Botryllus schlosseri* and *Drosophila* have been particularly instructive. In *Botryllus*, clusters of GSCs migrate through the vasculature toward the niche during asexual reproduction. However, *Botryllus* colonies can fuse with one another, and the resulting shared vasculature causes their GSCs to compete against each other [56,57,61]. Work has shown that the size of GSC clusters and the speed of their collective migration determine the winning GSCs. These traits are conserved within a GSC lineage, suggesting that they have a genetic basis that could be identified in the future. It is thought that Notch signaling may regulate cluster size [65], and additional future studies will identify other signals that regulate this process. It will also be important to determine what signals regulate collective migration, as well as the source of these signals. 

Studies in *Drosophila* ovaries have revealed that female GSCs with mutations in the differentiation-promoting gene *bam* outcompete wild-type GSCs as a result of upregulated autophagy, increased proliferation, and enhanced adhesion to the niche [81,82,83]. The role of Myc in GSC competition in the ovaries is still unresolved [81,82,85], which will be an interesting topic for future investigation. While many mutations have been shown to reduce fitness, causing GSCs to lose, it will be important to identify genes whose mutation allows a GSC to win. Within the *Drosophila* testis GSC competition model, there are multiple standing questions about *chinmo*-dependent GSC competition. In other stem cell competition models, winner cells will fill the niche space (a limited resource) vacated by the eliminated loser cells [29]. However, wild-type CySCs occupy this vacated space rather than *chinmo*-mutant GSCs [92]. It is possible that *chinmo*-mutant GSCs cannot outcompete neighbor CySCs for niche space, which is surprising given that de-differentiated spermatogonia have been previously shown to be able to outcompete CySCs [200]. Additionally, while *chinmo* is a known JAK-STAT target gene [201], STAT-depleted GSCs still express the Chinmo protein [153]. Therefore, regulators of Chinmo in male GSCs remain unknown. The study also found that ECM-related genes encoding Pcan, Dg, and βPS were upregulated in GSCs following depletion of Chinmo, but further investigation is needed to find out whether these are direct Chinmo target genes [63]. Identifying regulators and target genes of Chinmo in GSCs will be essential to further elucidate how *chinmo*-mutant GSCs outcompete their neighbors. It will also be important to conduct further studies to find out whether selfish germ cells with PAE-affiliated mutations or cancer stem cells use similar mechanisms to *chinmo*-mutant GSCs. 

More broadly, through clonal analyses, it will be important to identify other genes that confer competitive advantages to GSCs. One recent study reported that GSC clones with mutations in the lipase *brummer* grew to comprise a larger proportion of the GSC pool than control GSC clones [202]. It would be interesting to assess whether *brummer*-mutant GSCs derive their competitive advantage through the same mechanism as *chinmo*-mutant GSCs. Although technically cumbersome, performing a forward genetic screening in the ovaries or the testes for mutations that endow GSCs with enhanced fitness would be a powerful approach to identifying new regulators of GSC competition. Such research would also provide insights into whether there are additional mechanisms of GSC competition that do not involve niche remodeling in the testes or autophagy in the ovaries.

Studies of PAE disorders have demonstrated outcomes of GSC competition that have a clear impact on human health outcomes. As the age of a father increases, PAE-associated mutations become exponentially more likely to be found in the sperm, and these mutations cause a range of disorders associated with significant craniofacial and skeletal abnormalities [41]. However, much remains to be elucidated on this complex phenomenon. For example, while the consequences of mutations in the male germline can be considerable, the overall mutation rate in the male germline is low compared to somatic cell types [203,204]. This indicates that there are additional protective mechanisms for genomic integrity in these cells, and several possible mechanisms have been suggested [205,206]. It will be important in the future to identify these, which could inform our broader understanding of genome maintenance in the male germline.

Another open question in the field is how to generate in vivo models of PAE disorders. PAE disorders have not been found to naturally occur in mice, possibly because mouse SSCs do not seem to acquire DNMs over time like human SSCs [188]. While this phenomenon may be intriguing to investigate, it limits the potential for robust PAE disorder studies in an in vivo model. Notably, though, mouse SSCs with a PAE-associated mutation have been shown to have increased competitive ability [192]. Alternative models could also be explored in the future, such as human testicular organoids [207]. Finally, PAE disorders have been identified in part because they lead to severe phenotypes and are attributable to mutations in a single gene. It is possible that there are other PAE-associated mutations that lead to subtle phenotypes in offspring or require the presence of additional mutations to have an effect. These mutations would be challenging to study but could be beneficial to our understanding of the more nuanced effects of high paternal age.

In summary, cell competition is a robust and actively evolving field spanning many model organisms and tissue types. This field is consequential for our understanding of both basic cell biology and diseases such as cancer. GSC competition is particularly noteworthy because of its effect on the genotype of the next generation. Unanswered questions remain across all aspects of this field, especially regarding specific mechanisms of competition.

**Table 1:** Genes with a role in germline stem cell and somatic adult stem cell competition.

Gene	Organism/Tissue	Function	Role in Cell Competition	References
**Germline stem cells**				
*bag of marbles (bam)*	*Drosophila* ovary	Promotes differentiation of pre-cystoblast cells into cystoblasts	*bam*-mutant germline stem cell (GSC) clones upregulate autophagy leads and accumulate in the ovary stem cell niche. The role of E-Cadherin (E-Cad) in promoting the competitive abilities of *bam*-mutant GSCs is disputed.	[81,82,83]
*benign gonial cell neoplasm (bgcn)*	*Drosophila* ovary	Promotes differentiation of pre-cystoblast cells into cystoblasts	*bgcn*-mutant GSC clones upregulate E-Cad and force wild-type GSCs out of the ovarian stem cell niche.	[81]
*Myc*	*Drosophila* ovary	Growth-promoting pathway component	The role of *Myc* in competition is disputed. GSC clones with elevated Myc outcompete wild-type neighbors, suggesting that GSCs with lower Myc are replaced by those with higher Myc. However, *Myc*-null GSC clones are not outcompeted by wild-type GSCs.	[81,85]
*chronologically inappropriate morphogenesis (chinmo)*	*Drosophila* testis	Transcription factor which regulates neuronal temporal patterning; regulates eye development; maintains CySC sexual identity	*chinmo*-mutant GSC clones outcompete wild-type neighbors for niche access by forming an ECM ‘moat’ around the niche.	[92]
*FGFR2*	Human testis	Growth-promoting pathway component	The *FGFR2^S252W^* gain-of-function allele is linked to the paternal age affect (PAE) disorder Apert syndrome. *FGFR2* is presumed to have a role in SSC competition, but has not yet been tested in humans.	[179]
*FGFR3*	Human testis	Growth-promoting pathway component	The *FGFR3^G380R^* gain-of-function allele is linked to the PAE disorder achondroplasia. *FGFR3* is presumed to have a role in SSC competition, but has not yet been tested in humans.	[178]
*HRAS*	Human testis	RAS proteins downstream of several receptor tyrosine kinases (RTKs)	*HRAS* gain-of-function alleles are linked to the PAE disorder Costello syndrome. *HRAS* is presumed to have a role in SSC competition, but has not yet been tested in humans.	[182]
*PTPN11*	Human testis	Protein tyrosine phosphatase downstream of several RTKs	*PTPN11* gain-of-function alleles are linked to the PAE disorder Noonan syndrome. *PTPN11* is presumed to have a role in SSC competition, but has not yet been tested in humans.	[181]
*Fibroblast growth factor receptor (FGFR2)*	Mouse testis	Growth-promoting pathway component	Murine spermatogonial stem cell (SSCs) expressing the Apert syndrome *FGFR2^S252W^* gain-of-function allele have increased competitiveness in in vitro and in vivo models.	[192]
**Somatic adult stem cells**				
*hippo (hpo)*	*Drosophila* ovary	Tumor suppressor which negatively regulates Yorkie, the *Drosophila* homolog of YAP	*hpo*-mutant follicle stem cell (FSC) clones outcompete wild-type neighbor FSCs.	[106]
*hopscotch (hop)*	*Drosophila* ovary	Janus tyrosine kinase, part of JAK/STAT pathway	*hop*-over-expressing FSC clones outcompete wild-type FSCs.	[105]
*patched (ptc)*	*Drosophila* ovary	Hedgehog pathway component; tumor suppressor	*ptc*-mutant FSC clones outcompete wild-type FSCs.	[105]
*yorkie (yki)*	*Drosophila* ovary	Growth-promoting pathway component	FSC clones over-expressing *yki^S168A^*—a gain-of-function mutation—outcompete wild-type FSCs.	[106]
*Abelson (Abl)*	*Drosophila* testis	Kinase regulating growth, differentiation and adhesion	*Abl*-mutant cyst stem cell (CySC) clones outcompete wild-type neighbor CySCs.	[99]
*hippo (hpo)*	*Drosophila* testis	Tumor suppressor, negatively regulates Yorkie, the *Drosophila* homolog of YAP	*hpo*-mutant CySC clones outcompete wild-type neighbor CySCs.	[30]
*patched (ptc)*	*Drosophila* testis	Hedgehog pathway component; tumor suppressor	*ptc*-mutant CySC clones outcompete wild-type neighbor CySCs.	[30]
*Ras*	*Drosophila* testis	Kinase regulating growth	CySC clones over-expressing *Ras^GV12^*—a gain-of-function mutation—outcompete wild-type neighbor CySCs.	[96]
*Suppressor of cytokine signaling at 36E (Socs36E)*	*Drosophila* testis	Negative regulator of JAK/STAT and EGFR	*Socs36E*-mutant CySC clones outcompete wild-type neighbor CySCs.	[96]
*APC*	Mammalian intestine	Tumor suppressor	*APC*-mutant intestinal stem cell (ISC) clones outcompete wild-type ISCs, creating a monoclonal crypt.	[29]
*Kras*	Mammalian intestine	Kinase regulating growth	ISC clones over-expressing *KRAS^G12D^*—a gain-of-function mutation—outcompete wild-type ISCs, creating a monoclonal crypt.	[29]
*DNMT3A*	Human bone marrow	DNA methylation enzyme	*Dnmt3A*-mutant hematopoietic stem cells (HSCs) outcompete wild-type HSCs in competitive serial transplantation assays.	[144]
*Tet2*	Human bone marrow	DNA methylcytosine dioxygenase	*Tet2*-mutant HSCs outcompete wild-type HSCs in a competitive transplantation assay.	[145]
*Tp53*	Human bone marrow	Tumor suppressor	*Tp53* status does not impact HSC competition during homeostasis, but after DNA damage, *Tp53*^−/−^ HSCs have a competitive advantage over wild-type HSCs in mosaic animals.	[151]

**Table 2:** Cell competition-related genes associated with cancer.

Gene	Organism/Tissue	Function	Role in Cell Competition	References
*Jak2*	Human bone marrow	Tyrosine kinase in the JAK/STAT pathway	A gain-of-function mutation in human *Jak2^V617F^* causes bone marrow cells to outcompete wild-type cells via increased cell cycling in a competitive transplantation assay.	[122]
*RAS*	Mammalian epithelia in vitro and in vivo	Kinase regulating growth	Epithelial cells over-expressing *RAS^G12V^*—a gain-of-function mutation—are outcompeted by wild-type epithelia in EDAC.	[123]
*Tp53*	Mammalian epithelia in vitro and in vivo	Tumor suppressor	Epithelial cells expressing a dominant-negative Tp53 are outcompeted by wild-type epithelia in a process termed epithelial defense against cancer (EDAC).	[125]
*PIK3CA*	Mammalian esophagus	Catalytic subunit of phosphoinositide 3-kinase (PI3K)	Esophageal epithelial cells heterozygous for the gain-of-function mutation *Pik3CA^H1047R/+^* outcompete wild-type esophageal cells via biased cell fate toward proliferation.	[121]
*APC*	Mammalian intestine	Tumor suppressor	*APC*-mutant intestinal stem cell (ISC) clones cause wild-type ISCs to differentiate, thereby outcompeting them and leading to intestinal tumor initiation.	[44,45]
*YAP*	Mammalian intestine	Growth-promoting factor in the Hippo pathway	Epithelial cells over-expressing *YAP^5SA^*—a gain-of-function mutation—are outcompeted by wild-type epithelia in EDAC.	[124]
*YAP*	Mammalian liver	Growth-promoting factor in the Hippo pathway	Elevating YAP activity in hepatocytes through the 5SA mutation can drive liver tumor growth, if YAP activity is not higher in neighboring hepatocytes.	[126]
*Glial cell derived neurotrophic factor (GDNF)*	Mammalian testis	Secreted TGF-β ligand which is required for SSC niche maintenance	Misexpression in SSCs produces malignant tumors with germline markers.	[183]
